# Assessing Saudi women's knowledge of family planning methods: a cross-sectional study

**DOI:** 10.3389/fgwh.2026.1701619

**Published:** 2026-05-13

**Authors:** Tasneem Mohammed Makkawi, Abeer Saad Esawi, Shahrazad Mahmoud Timraz, Salah Eldin Makki Abbo

**Affiliations:** 1 Ministry of National Guard - Health Affairs, Jeddah, Saudi Arabia; 2 King Abdullah International Medical Research Center, Jeddah, Saudi Arabia; 3 College of Nursing, King Saud Bin Abdulaziz University for Health Sciences, Jeddah, Saudi Arabia; 4 College of Medicine, King Saud Bin Abdulaziz University for Health Sciences, Jeddah, Saudi Arabia

**Keywords:** contraception, cross-sectional, family planning, knowledge, Saudi women

## Abstract

**Introduction:**

Research on Saudi women's knowledge of family planning methods has emerged as a critical area of inquiry due to its impact on maternal and child health, population control, and women's empowerment. This study aimed to assess knowledge of family planning methods among Saudi women attending obstetrics/gynecology clinics.

**Methods:**

A descriptive cross-sectional study was conducted among 400 married Saudi women aged 18–49 years attending the Obstetrics and Gynecology Clinics at the National Guard Hospital, King Abdulaziz Medical City in Jeddah, between September and November 2024. Data were collected using a structured, self-administered questionnaire covering sociodemographic characteristics and knowledge of family planning methods.

**Results:**

Nearly half of participants (48.5%) were aged 25–34 years; 62.7% held a bachelor's degree, 59.2% were unemployed, and 70.8% reported prior use of family planning methods. Participants demonstrated a moderate overall knowledge (mean score 25.3 ± 9.3), with 47% classified as having moderate knowledge, 35.2% poor, and 17.8% good. Birth control pills (88.5%) and intrauterine devices (80.7%) were the most recognized methods; emergency contraception was the least known (21.7%). Family and friends (46.3%) were the most frequently cited information source, followed by health facilities (36%). Knowledge scores varied significantly by age, educational level, employment status, and prior family planning use in bivariate analyses (*P* < .05).

**Conclusion:**

Saudi women demonstrated moderate knowledge of family planning methods, independently associated with age, education, and prior use in adjusted analysis. These findings highlight the need to enhance women's informed use of family planning through reproductive health education in schools and community programs, personalized counseling during maternal care, and national policies that standardize counseling and ensure equitable access.

## 1 Introduction

Family planning (FP) is a cornerstone of reproductive health that empowers women and their partners to autonomously determine the number of children and the spacing of pregnancies (1). This is achieved through various contraceptive methods, including intrauterine devices (IUDs), sterilization, oral contraceptive pills, injections, and transdermal patches (1). In 2021, approximately 1.1 billion of the 1.9 billion women of reproductive age (15–49 years) worldwide expressed a need for FP services (1). Of these, 874 million were utilizing modern contraceptive methods, while 164 million had an unmet need for contraception due to limited availability of certain methods, age, income, marital status, fear of side effects, partner's consent, fear of stigma, and misinformation (1). Within this context, the unmet need for contraception among Saudi women is similarly driven by limited knowledge about reproduction and family planning, misconceptions, fear of side effects, inadequate counseling by healthcare providers, and social and cultural norms (2, 3). Access to FP methods plays a crucial role in preventing unintended pregnancies, reducing mortality rates, and mitigating associated health risks such as sexually transmitted diseases (STDs) (1, 4, 5). Additionally, some of the FP methods such as hormonal contraceptives are indicated for the treatment of endometriosis, uterine fibroids, heavy menstrual bleeding, polycystic ovary syndrome, acne, and migraines (4). For instance, a longitudinal study of 64 women with deep endometriosis (DE) and adenomyosis (AD) using flexible extended combined oral contraceptives (COC) demonstrated significant pain reduction and decreased sonographic evidence at the 12-month follow-up (6).

Saudi Arabia's Vision 2030 promotes women empowerment through education, workforce participation, and leadership opportunities (7), which may have influenced birth spacing and the number of children. Notably, the fertility rate among Saudi women declined from 3.8 in 2011 to 2.7 in 2024 (8). According to the United Nations (UN), the estimated prevalence of contraceptive use among women of reproductive age in Saudi Arabia was 18.6% in 2019, with the most reported methods being pills (11.1%) and IUDs (3.2%) (9). In 2024, 31.3% of Saudi married women reported using contraception, as indicated by the General Authority of Statistics in Saudi Arabia (10). Given the pivotal role of FP methods in promoting women's overall well-being, research examining women's knowledge of these methods within the Saudi Arabian context has increased in recent years. Some of these studies demonstrated inadequate and inconsistent knowledge of FP methods among Saudi women, which directly influences their proper utilization (3, 11–13). This gap between rising contraceptive use and inconsistent knowledge underscores the need for further investigation. Despite the expanding body of literature on FP in Saudi Arabia, existing studies have largely focused on oral contraceptives rather than providing a comprehensive assessment of knowledge across diverse FP methods. In addition, limited attention has been given to understanding how sociodemographic factors independently influence women's knowledge, thereby limiting a more nuanced and contextually grounded understanding of FP knowledge (11, 13–15).

In a cross-sectional study conducted among 1,107 Saudi women aged 18–45 in Al-Qunfudah, 50.2% reported using oral contraceptives (OC), primarily for FP purposes (81.1%). Nonetheless, knowledge of OC benefits was limited; 39.9% of non-users cited fear of side effects as a deterrent, while 48% expressed uncertainty regarding potential adverse effects. Notably, 73.0% of participants perceived the available contraceptive information from healthcare services as inadequate (11). Another cross-sectional study of 979 Saudi women aged 18 years and older in Jeddah found that 67.7% reported using contraceptives, primarily to prevent pregnancy (69.7%). OC (31.8%) and IUDs (21%) were the most used methods. Lower educational level was significantly associated with reduced contraceptive use, highlighting the need for targeted educational interventions and further research to address existing knowledge gaps (14). Conversely, other studies have reported satisfactory knowledge and positive attitudes toward FP methods (15, 16). This inconsistency underscores regional variations in women's knowledge across Saudi Arabia, which may contribute to unintended pregnancies, unmet contraceptive needs, and related health risks. Given the variability in reported knowledge levels and the increasing reliance on contraception in Saudi Arabia, understanding both the extent of knowledge and its associated factors is essential. Therefore, this study aims to assess FP knowledge among Saudi women and to determine sociodemographic characteristics associated with knowledge levels, to inform the development of targeted health promotion strategies, enhance patient education, and support informed decision-making in clinical settings.

## 2 Materials and methods

### 2.1 Study design

A cross-sectional analytical study design was employed to assess Saudi women's knowledge of family planning (FP) methods and to examine factors associated with knowledge levels at a single point in time.

### 2.2 Study setting and sampling

The study was conducted at the obstetrics and gynecology clinics of the national guard hospital, King Abdulaziz medical city, Jeddah, Saudi Arabia. These clinics provide a wide range of women's health services, making them an appropriate setting for assessing knowledge of FP methods.

A total of 400 participants were recruited through convenience sampling. The required sample size was calculated using the Raosoft® online calculator (17), with a 5% margin of error, a 95% confidence interval, and an assumed response distribution of 50%, yielding a minimum recommended sample of 384 participants. To ensure adequate representation and robustness, 400 women were ultimately recruited. Eligible participants were married Saudi women aged 18–49 years who attended the gynecology clinics during the study period. Women were excluded if they were non-Saudi nationals, 50 years or older, or if they reported using contraceptives primarily for the treatment of underlying medical conditions rather than for FP purposes.

### 2.3 Data collection

Data were collected using a structured, self-administered questionnaire adapted from Sey-Sawo et al. (18). The original instrument, developed in English, underwent a rigorous translation process involving forward translation into Arabic and subsequent back-translation into English to ensure linguistic accuracy, clarity, and cultural appropriateness. As the questionnaire was newly adapted for use within the Saudi context, establishing content validity was essential. Three midwifery experts independently evaluated each item using a four-point relevance scale. Most items achieved unanimous agreement, with an item-level content validity index (I-CVI) of 1.00, and the average scale-level content validity index (S-CVI) exceeding 0.90, indicating excellent overall validity.

The questionnaire was pilot-tested among 40 women (10% of the total study sample) to assess clarity, feasibility, and reliability. Since no major modifications were required, these participants were included in the final analysis.

The internal consistency of the adapted Arabic version of the family planning knowledge scale was 0.668. Although below the conventional 0.70 threshold, this value is acceptable in exploratory research and newly adapted cross-cultural instruments, where translation and contextual differences may influence response patterns (19, 20). Considering the multidimensional nature of the construct and the early phase of cultural adaptation, the obtained alpha value indicates satisfactory internal consistency for preliminary application. Item–total correlation analysis (*r* = 0.634–0.693) further supported the scale's internal coherence and provided insight for future refinement.

The questionnaire consisted of two main sections. The first section included multiple-choice questions that collected sociodemographic information such as age, educational level, employment status, region of residence, number of children, and prior use of FP methods. The second section assessed women's knowledge of FP using closed-ended, single-response items. This section included questions on the definition of FP (e.g., “What does family planning mean?”), awareness and familiarity with contraceptive methods (e.g., oral contraceptive pills, intrauterine device, condoms, and emergency contraception), and perceptions of their effectiveness. Additional items examined the benefits and importance of FP (e.g., prevention of unwanted pregnancy, promotion of maternal and child health, maintenance of birth spacing), contraindications (e.g., pregnancy, unexplained vaginal bleeding, ischemic heart disease, breast cancer), and side effects of hormonal methods (e.g., weight gain, mood changes, irregular bleeding, nausea, breast tenderness, and cancer risk), as well as warning signs requiring medical attention (e.g., severe abdominal pain, heavy bleeding, chest pain, blurred vision, or infection). The questionnaire also included items on appropriate sources of contraceptives (e.g., hospitals, family planning clinics, pharmacies), women's initial sources of FP information (e.g., family and friends, healthcare professionals, media), whether they had received prior formal education on FP, and their self-rated level of competence in using FP methods. Each correct response was scored as one point, with a maximum possible score of 54. Knowledge scores were categorized into three levels for descriptive interpretation: poor (0–20), moderate (21–35), and good (36–54).

### 2.4 Data analysis

Data were analyzed using the Statistical Package of Social Sciences (SPSS) software, version 29. Categorical variables were summarized using frequencies and percentages. The knowledge score, a continuous variable, was summarized using means and standard deviations. Bivariate comparisons between knowledge score and sociodemographic characteristics were conducted using independent-samples t-tests and one-way analysis of variance (ANOVA), as appropriate. Variables with theoretical relevance and/or statistical significance in bivariate analyses were entered into a multiple linear regression model to identify independent predictors of family planning knowledge. Regression coefficients (*β*), standard errors (*SE*), and *P*-values were reported. Statistical significance was set at *P* ≤ .05.

### 2.5 Ethical consideration

This study received ethical approval from the King Abdullah International Medical Research Center (KAIMRC) and the Institutional Review Board (IRB) of King Saud bin Abdulaziz University for Health Sciences, Jeddah (approval No. IRB/1222/24; dated 27 July 2024). Written informed consent was obtained from all participants after a clear explanation of the study objectives and procedures. The consent form emphasized that participation was entirely voluntary and that participants could withdraw at any time without penalty or obligation. Strict measures were applied to safeguard participants' anonymity and confidentiality. All documents containing identifiable data were securely stored in a locked cabinet separately from participants' responses, accessible only to the research team. Data will be retained for five years in accordance with institutional policies.

## 3 Results

### 3.1 Sociodemographic characteristics of participants

A total of 400 Saudi women participated in this study. The mean age of participants was 33.3 years (*SD* ± 4.7). Most participants were aged 25–34 years (48.5%), followed by those aged 35–44 years (32.3%). For educational attainment, 62.7% held a bachelor's degree, while 20.8% had completed secondary education or less. Regarding employment status, 59.2% of the participants were unemployed and 35.3% were employed, with most participants (86.8%) residing in the Western region of Saudi Arabia. In terms of parity, 24.5% reported having one child, 46.2% had two to four children, 13.5% had 5 or more children, and 15.8% had none. Notably, 70.8% of participants indicated previous use of family planning methods (Table 1).

**Table 1:** Sociodemographic characteristics of the participants (*N* = 400).

Variable	Frequency (n)	Percent (%)
Age		
18–24	36	9.0
25–34	194	48.5
35–44	129	32.3
≥45	41	10.2
Mean ± SD	33.3 ± 4.7	
Educational level		
Secondary or less	83	20.8
Diploma	36	9.0
Bachelor	251	62.7
Master	26	6.5
PhD	4	1.0
Employment status		
Student	22	5.5
Employed	141	35.3
Unemployed	237	59.2
Region of residence		
Central	34	8.5
Eastern	14	3.5
Madinah	5	1.2
Western	347	86.8
Number of children		
0	63	15.8
1	98	24.5
2–4	185	46.2
≥5	54	13.5
Ever use of family planning		
Yes	283	70.8
No	117	29.2

### 3.2 Knowledge of family planning methods

Participants demonstrated varying levels of understanding of the purpose of family planning (FP). The majority (64.3%) correctly identified FP as a process that supports decisions on when or whether to have children, including birth control options. A smaller proportion associated it solely with reproductive health education (45.2%), while only 21.5% mistakenly viewed it as a method for organizing family routines. Knowledge of specific FP methods varied considerably across participants. Birth control pills were the most recognized method (88.5%), followed by IUD (80.7%) and implants (65.7%). In contrast, knowledge of natural methods (36.7%) and injectable contraceptives (35.2%) was lower, with the least knowledge observed for emergency contraception (21.7%). Regarding perceived effectiveness, participants rated IUDs as the most effective method (52%), followed by birth control pills (42.7%), while injectable contraceptives were the least likely to be considered effective (9.2%). Participants identified “maintaining birth spacing” (73.7%) and “preventing unwanted pregnancy” (61.2%) as the most important benefits of FP methods. Preventing STDs (13.5%) and treating reproductive diseases (10.5%) were the least identified benefits. Regarding the perceived significance of FP use, participants mostly cited promoting maternal health (79.0%) and infant well-being (60.2%).

Regarding knowledge of FP contraindications, the most frequently reported conditions were current pregnancy (42.2%), unexplained vaginal bleeding (28.9%), and ischemic heart disease (22.2%). In terms of side effects, mood changes and weight gain (76.2%) and concern about cancer risk (55.2%) were commonly identified. Only 6% reported no side effects, while 11.2% indicated uncertainty. The most recognized warning signs were severe bleeding (44%) and abdominal pain (37.7%). In contrast, 31.7% of participants reported being uncertain about warning signs associated with the use of FP methods. Participants identified hospitals as the primary source of contraceptives (82.3%) followed by FP clinics (49.8%), and pharmacy (20.3%). Regarding sources of information about FP methods, family and friends were most frequently cited (46.3%), followed by health facilities (36%) and media (16.2%). Fewer than half of the participants (46.8%) reported receiving formal education on FP methods. Nevertheless, 42% rated their own knowledge as excellent, 33.3% as good, 19.7% as fair, and only 5% as poor. The mean knowledge score was 25.3 (*SD* ± 9.3), with scores ranging from 6 to 48. Almost half of the participants (47%) demonstrated moderate knowledge, 35.2% had poor knowledge and 17.8% had good knowledge of FP methods.

### 3.3 Association between sociodemographic characteristics and knowledge level of family planning methods

Significant associations were identified between knowledge of FP methods and several sociodemographic variables, including age (*P* = .001), educational level (*P* = .001), employment status (*P* = .001), and prior use of FP methods (*P* = .007). Higher knowledge levels were observed among participants aged 25–34 years, those with postgraduate education, employed women, and those who had previously used FP in unadjusted analyses (Table 2). In contrast, no significant differences were found based on region of residence or number of children (Table 2).

**Table 2:** Association between sociodemographic variables and family planning knowledge level.

Variable	Family Planning Knowledge Score		
	Mean	*SD*	*P*-value
Age			0.001^a^
18–24	19.1	7.4	
25–34	26.5	9.1	
35–44	26.3	9.7	
≥45	22.1	7.8	
Educational level			0.001^a^
Secondary or less	20.4	7.1	
Diploma	22.5	8.7	
Bachelor	26.7	9.4	
Postgraduate	30.7	9.6	
Employment status			0.001^a^
Student	23.1	8.9	
Employed	28.1	9.3	
Unemployed	23.8	9.0	
Region of residence			0.310
Central	23.1	9.2	
Eastern	24.1	10.2	
Western	25.5	9.3	
Number of children			0.993
0	25.6	8.9	
1	25.1	8.9	
2–4	25.3	9.5	
≥5	25.4	10.3	
Ever use of FP			0.007^a^
Yes	26.1	9.5	
No	23.3	8.6	

a Significant variable.

### 3.4 Predictors of family planning knowledge

Multiple linear regression analysis was conducted to identify independent predictors of family planning knowledge score. After adjusting for sociodemographic variables, age, educational level, and prior use of family planning were significantly associated with knowledge score. Participants aged 35–44 years demonstrated significantly higher knowledge scores compared with women aged 18–24 years (*β* = 2.44, *P* = .002). Similarly, women aged ≥45 years had higher knowledge scores (*β* = 1.72, *P* = .041). Although women aged 25–34 years showed higher scores than the reference group, the association did not reach statistical significance (*β* = 2.71, *P* = .052). Educational attainment was also significantly associated with knowledge. Women with a diploma (*β* = 3.75, *P* = .001), bachelor's degree (*β* = 2.41, *P* = .045), and postgraduate education (*β* = 1.52, *P* = .039) had significantly higher knowledge scores compared with women with secondary education or less. Furthermore, women who had previously used family planning methods demonstrated significantly higher knowledge scores than those who had never used such methods (*β* = 1.52, *P* = .005). Employment status, region of residence, and number of children were not significantly associated with knowledge score (Table 3).

**Table 3:** Multiple linear regression of knowledge score predictors.

Variable	*β*	*SE*	*P*-value
Age			
18–24 (Ref)	–	–	–
25–34	2.71	1.39	0.052
35–44	2.44	0.79	0.002
≥45	1.72	0.84	0.041
Educational level			
Secondary or less (Ref)	–	–	–
Diploma	3.75	1.01	0.001
Bachelor's	2.41	1.20	0.045
Postgraduate	1.52	0.73	0.039
Employment status			
Student (Ref)	–	–	–
Employed	0.17	1.41	0.903
Unemployed	1.03	0.91	0.261
Region of residence			
Central (Ref)	–	–	–
Eastern	−0.02	1.28	0.988
Western	−0.86	1.64	0.598
Number of children			
0 (Ref)	–	–	–
1	−0.32	1.08	0.764
2–4	−0.37	0.86	0.663
≥5	0.71	0.69	0.311
Ever use of family planning			
No (Ref)	–	–	–
Yes	1.52	0.53	0.005

## 4 Discussion

This study assessed knowledge of family planning (FP) methods among 400 Saudi women attending obstetrics and gynecological clinics at the National Guard Hospital in Jeddah. Although 70% of the participants reported prior use of FP methods, only 17.8% demonstrated good knowledge and 47% moderate knowledge. A similar trend was reported by Al Basri et al., who found that although 50.2% of married women of reproductive age used contraceptives, their knowledge of proper techniques and potential side effects was limited (11). These findings align with other studies in Saudi Arabia that consistently highlight poor FP knowledge among women (13, 21, 22). Moreover, a systematic review of 13 studies across the Kingdom confirmed that inadequate FP knowledge remains a persistent concern (12).

Regarding knowledge of specific FP methods, participants in this study were most familiar with birth control pills (88.5%) and IUDs (80.7%), which is consistent with findings reported in previous studies (13, 16). Alkalash et al. likewise found that in Al-Qunfudah, contraceptive pills (85.3%) and IUDs (57.8%) were the most recognized, whereas emergency and permanent methods were poorly known (23). Consistent with our results, emergency contraception was the least recognized method (21.7%). A study in the Eastern Province found that nearly three-quarters of women (73.8%) were unaware of the correct timeframe for its use (24). In contrast, Wali et al. reported that 61.6% of women had prior knowledge of emergency contraception, yet only 5% reported ever using it (25). This discrepancy between awareness and practice may be attributed to persistent misconceptions, inadequate counseling, and reliance on informal rather than professional sources of information.

Misconceptions about FP extend beyond the types and methods of use to include their side effects and potential benefits. In this study, more than half of the participants expressed concern about adverse effects such as mood changes, weight gain, and perceived cancer risk. These concerns mirror the limited knowledge and misconceptions identified in earlier research (21, 26). For example, Al Basri et al. reported that 39.9% of women avoided oral contraceptive pills (OCPs) due to fear of side effects (11), while another found that only 13.6% of women demonstrated adequate knowledge of OCPs, with weight gain and thrombosis being the most frequently cited risks (21). Participants' perceptions of FP benefits reflected limited awareness of their broader health implications. Although most recognized FP as a means of birth spacing and preventing unintended pregnancies, only a small proportion identified its role in preventing sexually transmitted diseases (13.5%) or managing reproductive health conditions (10.5%). In line with this, the study by Salem et al. reported that among 588 Saudi women, pregnancy prevention was the most acknowledged benefit of oral contraceptives, whereas acne reduction was the least recognized non-contraceptive advantage (21). Collectively, these findings suggest that health education and counseling efforts have largely emphasized pregnancy prevention and spacing, while insufficiently addressing the broader reproductive and non-contraceptive benefits of FP methods.

An additional noteworthy observation is the low rate of formal education on FP, reported by 46.8%, despite 42% of participants self-rating their knowledge as “excellent”. This discrepancy may suggest overconfidence or reliance on informal information sources. A large proportion of participants cited family and friends (46.3%) as primary sources of FP knowledge, followed by health facilities (36%), while social media was the least reported source (16.2%). Literature shows mixed results; some studies confirm similar trends (21, 23), whereas others highlight the internet and social media as more dominant sources of information (25, 27, 28). These discrepancies underline the limitations of non-professional sources, which often perpetuate misinformation and reinforce misconceptions about FP methods. Importantly, this reliance on informal channels is not unique to Saudi Arabia; global evidence shows similar pattern, where misinformation thrives when professional guidance is limited (29, 30).

Sociodemographic factors were significantly associated with FP knowledge in both bivariate and multivariable analyses. In the adjusted regression model, age and educational level emerged as independent predictors of knowledge score. Specifically, women aged 35–44 years and those aged ≥45 years demonstrated significantly higher knowledge scores compared with the youngest age group (18–24 years), while the difference for the 25–34 age group did not reach statistical significance. Similarly, higher educational attainment was associated with greater knowledge, with diploma, bachelor's, and postgraduate graduates all scoring significantly higher than those with secondary education or less. Prior use of family planning methods was also independently associated with higher knowledge. Employment status, by contrast, was significantly associated with knowledge in bivariate analysis but did not remain an independent predictor after adjustment for other sociodemographic variables. These findings mirror those reported in a cross-sectional study involving 672 Saudi participants, where age, educational level, and working status were identified as strong predictors of FP awareness (27) and are consistent with comparable associations reported both within and outside Saudi Arabia (16, 21, 23, 29–31). Collectively, these results underscore the importance of tailoring FP education to demographic characteristics and ensuring broader dissemination of accurate, evidence-based information, particularly among younger and less-educated women, to counter persistent misconceptions and enhance effective utilization of family planning services.

### 4.1 Limitations

This study has some limitations that must be considered when interpreting its findings. First, participants were exclusively recruited from a single tertiary healthcare setting through convenience sampling, which may have introduced selection bias. It is possible that women attending such facilities differ from those in community or primary-care settings in terms of educational attainment, socioeconomic status, or exposure to health information, which could have influenced their knowledge levels. Consequently, the findings may not fully represent women from other socioeconomic or regional contexts. Future studies should therefore employ multi-site recruitment strategies across primary, secondary, and community healthcare facilities to improve representativeness and enhance the generalizability of results. Second, the cross-sectional design limits the ability to establish cause-and-effect relationships. Third, self-reported data are subject to recall bias, which may have influenced participants' responses regarding their knowledge and use of family planning methods.

### 4.2 Implications

The findings of this study underscore the urgent need to strengthen women's knowledge and informed use of FP methods as a cornerstone of reproductive and public health advancement. Improving family planning awareness through formal and informal education can foster early understanding and more positive attitudes toward FP use. Efforts to integrate reproductive health education within schools, universities, and community programs will help normalize discussion about family planning and empower women and younger generations to make informed reproductive choices. In clinical settings, consistent and individualized counseling during maternal and postpartum care is vital to support women's decision-making and ensure continuity of care. At the structural level, national frameworks that standardize counseling practices, promote equitable access to contraceptive methods, and include measurable performance indicators are essential to sustain progress and align reproductive health services with the national Vision 2030 objectives for women's empowerment and health equity.

### 4.3 Recommendation

Drawing from these implications, a coordinated and multidimensional approach is recommended to enhance women's knowledge, access, and informed utilization of family planning services. Educational institutions should embed reproductive health and family planning content within curricula, supported by culturally relevant and community-based awareness initiatives that reach women of diverse educational and socioeconomic backgrounds. Healthcare providers must receive ongoing professional development to refine counseling skills, strengthen cultural competence, and improve the clarity and accuracy of communication with patients and their families. Policy leaders should ensure the establishment and enforcement of national guidelines for family planning counseling, allocate sufficient resources to sustain outreach and service delivery, and maintain transparent monitoring systems that track progress and accountability. Future research should adopt multi-site sampling to capture broader perspectives across different healthcare and community contexts and consider the Health Belief Model as a guiding framework to explore how women's perceptions influence their family planning decisions. Collectively, these measures can advance the quality, accessibility, and sustainability of FP services, contributing to healthier families, empowered women, and a more equitable healthcare system.

## Data Availability

The raw data supporting the conclusions of this article will be made available by the authors, without undue reservation.
